# Supplementary material for: Development, factor structure and application of the Dog Obesity Risk and Appetite (DORA) questionnaire
Source: PeerJ. 2015 Sep 29;3:e1278. doi: 10.7717/peerj.1278 (PMC4592153; doi:10.7717/peerj.1278)
Supplement: File S1 [file peerj-03-1278-s002.pdf]

Supplementary file 1: Primer used during semi-structured interviews.

Introduction: My name is ER and I'm the vet running the GOdogs project at the U of C. Thank you for volunteering for our study. I'd really like to hear a bit about ___, in particular his/her attitude to food.

Next, start a deliberately unstructured part of the conversation, asking the owner to volunteer information. Usually prompted by asking the following:

'Perhaps you can start by telling me what ___ is like with food?'

And later:

'Why do you think ___ is the size and shape he/she is?'
'How long have you had ___?'
'What else goes on for ___ in every day – daily life, exercise and so on?'

The owner's responses should be used as a guide for the subsequent conversation, and to ensure the **following topics were covered. Example questions are included for each topic.**

	Topic	Example Questions
Dog Topics		
a	Responsiveness to food	How do you feed ___? What happens at feeding time?<br>Describe how ___ eats<br>What's ___ like once the food is finished?<br>What do you feed ___?<br>Is ___ choosy about different foods?<br>Does he/she get any titbits or human food? What, and how often? What's ___ like when offered food between his/her usual meals?<br>What's ___ like when you are preparing human food?<br>What's ___ like when you are eating?<br>Do you have other dogs? Have you had other dogs in the past? How does ___'s attitude to food compare?
b	Speed of eating	
c	Satiety responsiveness	
d	General interest in eating	
e	Food fussiness	
f	Emotional eating	Does ___ ever go off food? If so, why? What about when you go away from home?<br>Does food help when he/she is frightened or upset?<br>How useful is food as a training aid or reward?
g	Scavenging/stealing food	Does he/she steal food? Dog food? Other pets' food? Human food?<br>What's he/she like when he/she comes across discarded human food or disgusting stuff on a walk?<br>Does ___ seek out food when out on walks or in the garden?
h	Food related aggression	What's he/she like when there's food around? Does ___ get grumpy around food?

Owner Management Topics		
a	Control over eating	See a-d above.<br>Do you measure how much you feed? How?<br>Do you interfere if ___ finds something edible out and about?
b	Prompting/encouragement	See a-d above.<br>Do you do anything to make food more interesting? Do you ever change ___'s diet? Why?<br>Do you have to encourage ___ to eat? How?
c	Instrumental feeding	Do you use food rewards in training? Did you in the past? How helpful are/were they?
d	Emotional feeding	See (f) above.
e	Importance of body shape	Are you happy with ___ weight? Do you think he/she could do with losing or gaining any weight?<br>What do you do to maintain that weight? What do you do to alter it? How important is it to keep a dog at a healthy weight?
f	Exercise	What sort of execise does ___ get? Do you/how do you play with your dog? Does he/she work or do agility? Does he/she match a human exercise programme?<br>How long do you spend taking your dog out each day? How does that vary through the week/year?
g	Level of training	Does he/she work or do agility?<br>What's his/her personality like?<br>How well trained is your dog? How do you think it may influence your dog's behaviour around food, if at all?<br>How well can you control behaviour around food that you don't like (like scavenging)?
h	Health	Tell me about his/her health. Any one off or recurrent problems over the years? Is he/she on any long term treatment or has he/she been in the past?<br>Does ___ ever get tummy upsets? Are there any things he/she can't eat?

Conclusion:
Do you have any questions for me?
Thank you; explain how to find out more about the project and future results; discuss whether owner would mind being contacted in future.
