# Supplementary material for: Development, factor structure and application of the Dog Obesity Risk and Appetite (DORA) questionnaire
Source: PeerJ. 2015 Sep 29;3:e1278. doi: 10.7717/peerj.1278 (PMC4592153; doi:10.7717/peerj.1278)
Supplement: File S2 [file peerj-03-1278-s003.pdf]

Dog Obesity Risk Assessment Questionnaire

This document has been prepared to give an overview of how the questionnaire was presented to owners in the current study (Raffan et al 2015). Since the original was administered via a commercially available online software package, it is not entirely representative of the format presented to owners, but gives a good indication of what they saw and the representative screenshot below shows how it appeared online. **BOLD CAPITALS** indicate the order of items which appeared on the website to owners, but do not form the core questionnaire.

Representative screen-shot of questionnaire displayed in web browser:

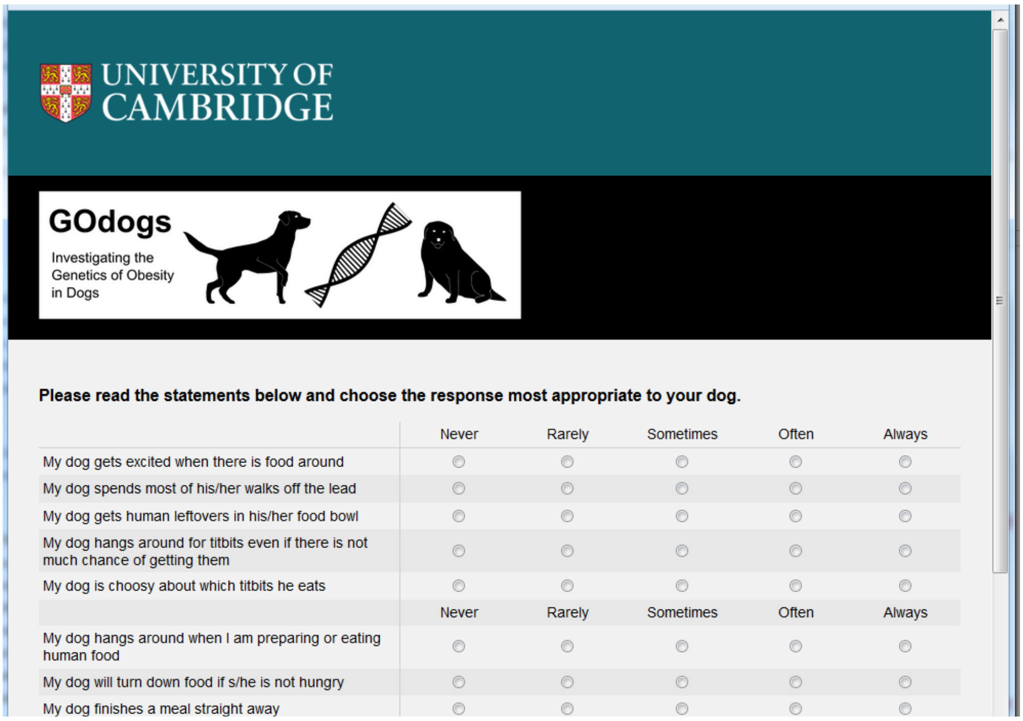

PROJECT LOGO AND UNIVERSITY OF CAMBRIDGE LOGO

NAMES AND CONTACT DETAILS OF STUDY INVESTIGATORS

INTRODUCTION:

The GOdogs project is investigating the genetics of obesity and appetite in dogs. Our overall aim is to improve dog and hopefully human health by understanding obesity and how to prevent and treat it. We have developed this short questionnaire as a way of scoring dogs' attitudes to food so we can compare individual dogs' and dog breeds' attitudes to food. It takes 5-10 minutes to complete and we would be extremely grateful if you would take the time to answer the questions.

QUESTIONS TO COLLECT INFORMATION ABOUT DOG SIGNALMENT AND OWNER CONTACT (VOLUNTARY)

NEW WEBPAGE

CORE DORA QUESTIONNAIRE SECTION (TWO QUESTIONS A AND B BELOW)

A) Please read the statements below and choose the response most appropriate to your dog.

	Never	Rarely	Sometimes	Often	Always
My dog gets excited when there is food around	❍	❍	❍	❍	❍
My dog spends most of his/her walks off the lead	❍	❍	❍	❍	❍
My dog gets human leftovers in his/her food bowl	❍	❍	❍	❍	❍
My dog hangs around for titbits even if there is not much chance of getting them	❍	❍	❍	❍	❍
My dog is choosy about which titbits he eats	❍	❍	❍	❍	❍
My dog hangs around when I am preparing or eating human food	❍	❍	❍	❍	❍
My dog will turn down food if s/he is not hungry	❍	❍	❍	❍	❍
My dog finishes a meal straight away	❍	❍	❍	❍	❍
My dog inspects unfamiliar foods before deciding whether to eat them	❍	❍	❍	❍	❍
My dog runs around a lot	❍	❍	❍	❍	❍
After a meal my dog is still interested in eating	❍	❍	❍	❍	❍
My dog takes his/her time to eat a meal	❍	❍	❍	❍	❍
My dog eats titbits straight away	❍	❍	❍	❍	❍
My dog gets bits of human food when we are eating	❍	❍	❍	❍	❍

NEW WEBPAGE

B) As before, please read the following statements and choose the option most appropriate to your dog. The headings for the answers have changed for this section.

	Not at all true	Somewhat true	Mainly true	Definitely true
My dog would eat anything	❍	❍	❍	❍
My dog is very fit	❍	❍	❍	❍
My dog often gets human food	❍	❍	❍	❍
My dog gets an upset tummy on some foods	❍	❍	❍	❍
I think my dog could do with losing some weight	❍	❍	❍	❍
My dog's walks are mostly on the lead	❍	❍	❍	❍
I restrict my dog's exercise because of veterinary advice	❍	❍	❍	❍
I alter the food my dog gets in order to control his/her weight	❍	❍	❍	❍
My dog seems to be hungry all the time	❍	❍	❍	❍
My dog's walks involve a lot of energetic play or chasing	❍	❍	❍	❍
I am careful about my dog's weight	❍	❍	❍	❍
My dog has a sensitive stomach	❍	❍	❍	❍
My dog is very greedy	❍	❍	❍	❍
My dog regularly sees the vet for health problems	❍	❍	❍	❍
I am happy with my dog's weight	❍	❍	❍	❍
I weigh or measure how much food I give my dog	❍	❍	❍	❍
I am careful to regulate the exercise my dog gets in order to keep him/her slim	❍	❍	❍	❍
My dog gets a lot of exercise	❍	❍	❍	❍
My dog often gets tummy upsets	❍	❍	❍	❍
My dog gets no food at human mealtimes	❍	❍	❍	❍
My dog would eat non-food objects like stones, toys or socks.	❍	❍	❍	❍

NEW WEBPAGE

BODY CONDITION CHART AND SCORE SELECTION

TWO COMMENTS BOXES (FREE TEXT)

A) **Use this box to tell us anything about your dog's eating behaviour, weight, or management that you think is important.**
B) **This is a pilot survey - the final version will include only a small sample of the questions you have patiently answered. Please let us know here if there are any questions you found hard to answer.**

‘CAPTCHA’ (Completely Automated Public Turing test to tell Computers and Humans Apart)

NEW WEBPAGE

THANK YOU, CONSENT STATEMENTS, REPEAT OF PROJECT AND INVESTIGATOR CONTACTS
